# Predictors of health worker performance after Integrated Management of Childhood Illness training in Benin: a cohort study

**DOI:** 10.1186/s12913-015-0910-4

**Published:** 2015-07-21

**Authors:** Laura C. Steinhardt, Faustin Onikpo, Julien Kouamé, Emily Piercefield, Marcel Lama, Michael S. Deming, Alexander K. Rowe

**Affiliations:** Malaria Branch, Division of Parasitic Diseases and Malaria, Centers for Disease Control and Prevention, Atlanta, GA USA; Direction Départementale de la Santé Publique de l’Ouémé et Plateau, Ministry of Public Health, Porto Novo, Benin; Africare-Benin, Porto Novo, Benin; Parasitic Diseases Branch, Division of Parasitic Diseases and Malaria, Centers for Disease Control and Prevention, Atlanta, GA USA

**Keywords:** Health worker performance, Case management, IMCI, Benin, Quality of care, Child health

## Abstract

**Background:**

Correct treatment of potentially life-threatening illnesses (PLTIs) in children under 5 years, such as malaria, pneumonia, and diarrhea, can substantially reduce mortality. The Integrated Management of Childhood Illness (IMCI) strategy has been shown to improve treatment of child illnesses, but multiple studies have shown that gaps in health worker performance remain after training. To better understand factors related to health worker performance, we analyzed 9,330 patient consultations in Benin from 2001–2002, after training one of the first cohorts of 32 health workers in IMCI.

**Methods:**

With data abstracted from patient registers specially designed for IMCI-trained health workers, we examined associations between health facility-, health worker-, and patient-level factors and 10 case-management outcomes for PLTIs.

**Results:**

Altogether, 63.6 % of children received treatment for all their PLTIs in accordance with IMCI guidelines, and 77.8 % received life-saving treatment (i.e., clinically effective treatment, even if not exactly in accordance with IMCI guidelines). Performance of individual health workers varied greatly, from 15–88 % of patients treated correctly, on average. Multivariate regression analyses identified several factors that might have influenced case-management quality, many outside a manager’s direct control. Younger health workers significantly outperformed older ones, and infants received better care than older children. Children with danger signs, those with more complex illnesses, and those with anemia received worse care. Health worker supervision was associated with improved performance for some outcomes.

**Conclusions:**

A variety of factors, some outside the direct control of program managers, can influence health worker practices. An understanding of these influences can help inform the development of strategies to improve performance.

**Electronic supplementary material:**

The online version of this article (doi:10.1186/s12913-015-0910-4) contains supplementary material, which is available to authorized users.

## Background

Although much progress has been made in the last decade, 7.6 million children worldwide still die each year before their fifth birthday, and half of the 4.5 million deaths among children between one and 59 months old are due to pneumonia, diarrhea, and malaria [1]. These three diseases all have effective treatment that can be delivered within community- or facility-based settings [2–4]. Evidence from field studies of routine care support the importance of correct case management for preventing child deaths, with an analysis of outpatient visits for sick children in western Kenya finding that appropriate health facility-based treatment reduced mortality by 78 % [5].

The Integrated Management of Childhood Illness (IMCI) strategy was designed to address the major causes of childhood mortality in a comprehensive manner and was first introduced the 1990s [6, 7]. IMCI has been recommended by the World Health Organization (WHO) and the United Nations Children’s Fund (UNICEF) and implemented in more than 100 low- and middle-income countries [8]. As the symptoms of childhood illnesses can be non-specific and overlapping, with co-morbidities common, IMCI provides an algorithmic framework for systematically assessing, classifying, and treating common childhood illnesses [9]. The cornerstone of IMCI, along with strengthening health system supports and community health practices, is improving the skills of facility-based health workers through IMCI training, an 11-day course focused on syndrome-based management of children for identifying and treating illnesses. Evaluations of IMCI have found mixed results, with improvements in disease classification, treatment, and counseling in several countries [10, 11], but inadequacies in overall implementation, health system supports and community guidelines and practices [9, 12].

Despite significant improvements in quality of care after IMCI training, important performance gaps remain, with some studies indicating that about one-third of sick children still do not receive correct treatment for potentially life-threatening conditions after health workers receive IMCI training [11, 13]. Significant decreases in mortality after IMCI training have been found in a few studies [11, 14–18] but not in others [14, 15], likely due to both low use of the formal health care system, lack of health system infrastructure, and gaps in health worker performance.

To help close gaps in case-management quality, it is important to understand more fully the factors influencing health worker performance, but definitive evidence on determinants of health worker performance is lacking [19]. We had a unique opportunity to examine an unusually large number of outpatient consultations performed in Benin by IMCI-trained health workers, who were part of a larger trial of additional health worker supports after IMCI training. Drawing from more than 9,000 register records of consultations with children ages 2–59 months, we were able to examine multiple treatment outcomes, including three cross-cutting quality of care outcomes and seven disease-specific outcomes. Whereas previous analyses have typically focused on a single disease outcome (e.g., malaria), this analysis of 10 outcomes allowed us to assess factors that were consistently related to case-management quality across a range of potentially life-threatening illnesses (PLTI). This approach enabled us to determine the most important predictors of health worker performance for treatment of child illnesses. A previous analysis using this dataset focused on the trends in health worker performance over time following initial IMCI training [21] but not on other predictive factors. This analysis focuses on a wide array of other factors that might influence health worker practices. Although the IMCI guidelines have been updated since the time of data collection in 2001–2002, it is still relevant and informative to understand the reasons behind health worker non-adherence to guidelines, a problem that persists today [22, 23].

## Methods

### Study setting and data source

We used data from a larger trial of supports for IMCI-trained health workers in Ouémé and Plateau Departments, in southeastern Benin. After the 11-day IMCI training, health workers in the trial’s intervention arm received additional job aids, including modified IMCI patient registers, a counseling guide, non-financial incentives in the form of framed ‘certificates of merit’ given to health workers at a ceremony every 1–2 years, and strengthened supervision visits focused on IMCI [20]. The IMCI patient registers were designed as job aids to facilitate comprehensive patient care, and had spaces for health workers to record details on the patient’s assessment, disease classifications, treatments prescribed, and any referrals made (see Additional file 1: Web Appendix 1). From details in the registers, we could essentially recreate patient consultations to examine the extent to which health workers followed IMCI guidelines.

Data for this analysis were abstracted from IMCI registers for initial consultations (first visit to the health facility for the disease episode) among children ages 2–59 months seen by a cohort of the first 32 IMCI-trained health workers in the intervention arm of the aforementioned trial. For each health worker, records were abstracted for 12 months after the 11-day IMCI training, which occurred in several rounds from June–October 2001, and were double-entered and verified with EpiInfo version 6 (CDC, Atlanta, Georgia). For most of the register abstraction study period (June 30, 2001–November 14, 2002), all consultations were abstracted; towards the end of the study period, a systematic sample of consultations was selected, due to limited resources. Additional details on the methodology of the record review have been described elsewhere [21].

### Analysis

We included consultations with at least one PLTI and used a computer program to apply IMCI guidelines to patients’ clinical findings documented in the registers to create a gold standard set of IMCI classifications and treatments for each consultation. We analyzed ten case-management outcomes: three primary and seven secondary (Table 1 and Box 1).

**Table 1:** Descriptions and levels of health worker performance for various measures of case management quality

Indicators of case management quality	Description	*N*	Mean (%)
*Primary outcomes*			
1. Recommended treatment for all potentially life-threatening illnesses (PLTIs)	Essential treatment of all PLTIs a child had (including malaria, very severe febrile illness, pneumonia, severe pneumonia, diarrhea, anemia, and severe anemia) that exactly matched IMCI guidelines†	8,277	63.6
2. Adequate treatment for all PLTIs	Essential treatment of all PLTIs considered effective^a^ even if it did not exactly match IMCI guidelines	8,277	77.8
3. Percentage of all needed tasks performed per child (index of overall guideline adherence)	Percentage of total IMCI-recommended assessment tasks (maximum of 39), disease classification tasks (maximum of six), and treatment tasks (maximum of four) done (see Box 1 for details)	9,330	96.5
*Secondary outcomes*			
4. Recommended treatment for febrile illnesses	First- or second-line antimalarial (chloroquine, sulfadoxine-pyrimethamine, respectively) for malaria or injectable quinine plus referral for very severe febrile illness, with dosages that exactly match IMCI guidelines	6,872	80.1
5. Adequate treatment for febrile illnesses	Antimalarial and dose considered effective even if it did not exactly match IMCI guidelines	6,872	87.5
6. Recommended treatment for anemia	Antimalarial, iron, and folate all given for anemia (with dosages that exactly match IMCI guidelines) or referral for severe anemia	3,133	47.1
7. Adequate treatment for anemia	Anemia treatments all given with doses considered effective even if they did not exactly match IMCI guidelines	3,133	75.6
8. Recommended treatment for pneumonia	First- or second-line antibiotics (cotrimoxazole, amoxicillin, respectively) for pneumonia or injectable ampicillin plus referral for severe pneumonia, with dosages that exactly match IMCI guidelines	2,787	76.0
9. Adequate treatment for pneumonia	Antibiotic and dose considered effective even if it did not exactly match IMCI guidelines	2,787	82.7
10. Recommended treatment for diarrhea	Oral rehydration solution given, plus antibiotic for bloody diarrhea, with antibiotic dosages that exactly match IMCI guidelines	1,680	81.6

Note: Analysis excludes consultations that took place on the day of the IMCI follow-up visit, as these may not be representative of typical health worker performance

† Includes referral for hospitalization for severe illness

^a^That is, life-saving, based on standard clinical textbooks (Gilbert *et al.* 2004; Robertson & Shilkofski 20015). Adequate treatment could have included errors such as small, clinically insignificant overdoses

Nine outcomes were dichotomous, and one was continuous—an index from 0 to 100 % of the percentage of needed IMCI tasks that was performed per patient (Box 1). Statistical analysis was carried out in SAS 9.3 (SAS Institute, Inc., Cary, North Carolina). We performed logistic regression on binary outcomes using the SAS SURVEYLOGISTIC procedure, and linear regression on the continuous outcome using the SURVEYREG procedure. Both procedures use the Taylor linearization series to account for clustering, in this case at the level of the health worker, and weighting, as a small proportion of the record reviews were sampled towards the end of data abstraction.

For each outcome, we systematically analyzed its relationship with factors at various levels (patient, health worker, and health facility), first using bivariate and then multivariable analysis. We included a broad range of plausibly related factors, as our analyses were exploratory in nature. We drew upon previous literature, as well as plausibility of effects, to determine which factors to test for their relationship to the outcomes. Factors with a p-value < 0.10 from the bivariate logistic/linear regression models were retained for inclusion in multivariable regression models. For each outcome, we tested four *a priori* interactions: 1) number of IMCI-trained workers at the facility and number of prior supervisory visits by the time of consultation, 2) health worker age and number of prior supervisory visits, 3) health worker age and caseload on the day of the child’s visit, and 4) child’s temperature and child’s age. Interaction terms were included in the model if they were statistically significant and remained so in a stable model after adding other covariates. Collinearity of the model was assessed, and variables were removed if warranted. Covariates that were not significant at the p < 0.10 threshold in bivariate analysis were then added back into the model one at a time, by ascending order of their p-value in bivariate analysis. Added covariates were retained in the model as confounders if they changed the measure of association (usually an odds ratio) of any other variable in the model by 20 % or greater, except if the originally included variables were not initially significant at the p < 0.05 level and remained non-significant after the addition of the potential confounder. Added variables were retained in the model as predictors if their p-value was <0.05. Each time a new variable was added to the model, the process of checking confounders/additional predictors was repeated in an iterative manner until all covariates had been assessed for the current model. Although multiple comparisons were made, given that we had 10 outcomes, no adjustments for significance levels were made, but we considered factors to be key predictors only if they were significant (in the same direction) at the p < 0.05 level across at least two outcomes.

### Ethical approval

The protocol for the larger trial of IMCI supports was approved by the Ethics Committee of the Benin Ministry of Public Health and the Centers for Disease Control and Prevention’s Human Subjects Review Board. All health workers provided written, informed consent to participate in the study.

## Results

The 32 IMCI-trained health workers were all nurses working at 21 different health facilities: 14 smaller primary care clinics and 7 larger referral facilities. Altogether 13,324 visits were recorded in the 12 months following IMCI training, and of these 9,402 initial consultations were abstracted. Nine initial consultations were excluded because of implausible values, and 63 were excluded because they occurred on the day of a supervisor’s post-IMCI training follow-up visit (about four to six weeks after the IMCI course) and were not thought to be representative of typical consultations. The remaining 9,330 initial consultations were screened for eligibility in the analysis. Each health worker had a median of 252 consultations in the dataset (range: 31–704). As a measure of data completeness, we calculated that 98.4 % of required IMCI assessment tasks were documented by health workers in the registers.

Among the 9,330 initial consultations, 8,277 (88.7 %) involved a PLTI according to gold standard IMCI algorithms, including malaria/fever (6,872, or 73.6 %), anemia (3,133 or 33.6 %), pneumonia (2,787 or 29.9 %), and diarrhea (1,680 or 18.0 %) (Table 1 and Box 1). Among children with a PLTI, 41.0 % had one PLTI, 41.4 % had two, 16.3 % had three, and 1.3 % had four.

**Fig. 1:**
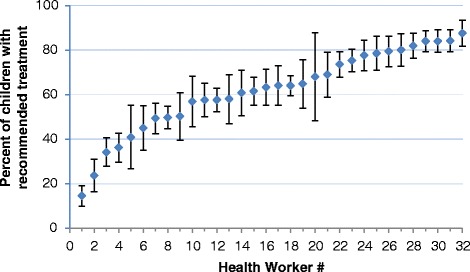
Graph of recommended treatment by individual health worker. Note: Vertical lines represent confidence intervals

For children with at least one PLTI, 63.6 % were treated exactly according to IMCI guidelines, and 77.8 % were treated adequately (i.e., effective treatment even if it did not exactly match IMCI guidelines) (Table 1 and Box 2). Performance varied widely by health worker, ranging from an average of 15 % to 88 % of children receiving recommended treatment (Fig. 1). The mean percentage of total IMCI tasks (assessment, classification, and treatment) done per patient was quite high, at 96.5 %. This result was primarily driven by the large number of assessment tasks (maximum of 39) that were very thoroughly performed, and by the generally correct classifications (mean of 93.2 %), despite much lower levels of correct treatment (74.2 %).

The provision of recommended treatments for febrile illness, pneumonia, and diarrhea was reasonably good, at 80.1 %, 76.0 %, and 81.6 %, respectively (Table 1). Adequate treatment, which included recommended treatment plus treatment that deviated from the IMCI guidelines but was still lifesaving, for febrile illness and pneumonia was six to seven percentage points higher than recommended treatment. Recommended treatment for anemia, however, (which required correctly dosed iron, folate, and an antimalarial) was substantially lower, at 47.1 %. Adequate treatment of anemia was 75.6 %.

One-third of consultations analyzed took place at large primary care facilities (Table 2). Children were seen by health workers with a mean age of 36 years (range = 25, 53), and approximately three-quarters were seen by female health workers. Patient volumes (in terms of children 2–59 months old seen per day) were relatively low at the included health facilities, with only 6.5 % of consultations taking place on days when the health worker saw at least 10 children in this age range. Health workers almost certainly performed consultations on patients outside the 2–59 month range, however caseload data for these patients were not abstracted. About one-fifth of consultations were performed by health workers who had received specific malaria training prior to their IMCI training. Children were seen by health workers who had received on average 1.6 supervision visits in the last six months. About half of children were seen in a health facility in which at least one other IMCI-trained health worker was present.

**Table 2:** Health facility and health worker characteristics

Variable	Mean (%)
	*N*=8,277 consultations
*Health facility level*	
Large (vs. small) facility (district-level referral center vs. clinic)	33.3
*Health worker level*	
Health worker age in years , mean (range)	35.8 (25, 53)
Female health worker	73.5
10+ consultations on day of child's visit^a^	6.5
Previous malaria training^b^	20.5
# supervision visits in previous 6 months, mean (range)^†^	1.6 (0, 8)
1+ other IMCI-trained health worker at facility^†^	50.6

Note: for consultations with at least one potentially life-threatening illness

^a^Consultations of patients 2–59 months

^b^Prior to IMCI training, which all health workers received. Previous diarrhea training (25.6 %) instead of previous malaria treatment was modeled for diarrhea treatment outcome

^†^Values may vary over time for the same health worker

Forty percent of consultations were for infants between 2 and 11 months old (Table 3). The mean temperature among children was 38° Celsius, and children required on average 26.5 IMCI tasks to be performed (for assessment, classification, and treatment), with a range from 18 to 43. Danger signs, including unconsciousness, lethargy, inability to eat or drink, vomiting everything, and history of convulsions, were rare, present in only 2.7 % of recorded consultations. The most common classifications, according to gold-standard classifications derived from assessment findings, were fever (86.2 %), anemia (38.3 %), pneumonia (34.0 %), and diarrhea (18.6 %). The most common documented patient complaints to the health worker were fever (75.2 %), cough or difficult breathing (39.8 %), and diarrhea (15.3 %) (Table 3).

**Table 3:** Patient characteristics

Variable	Mean (%)
	*N*=8,277 consultations
Age <1 year	40.0
Female sex	45.4
Patient temperature in degrees Celsius, mean (range)	38.0 (31.0, 41.7)
# total IMCI tasks required (case complexity)	26.5 (18, 43)
At least one danger sign	2.7
Gold-standard classification of fever^a^	86.2
Gold-standard classification of anemia^a^	38.3
Gold-standard classification of pneumonia^a^	34.0
Gold-standard classification of diarrhea^a^	18.6
Patient complaint of cough or difficult breathing	39.8
Patient complaint of diarrhea	15.3
Patient complaint of fever or malaria	75.2

^a^Derived from computer algorithm analysis of documented patient assessment variables

**Fig. 2:**
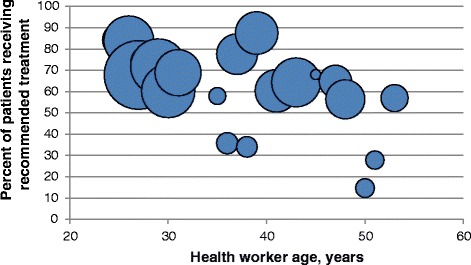
Bubble plot graph of health worker age and recommended treatment. Note: Size of bubbles proportional to number of consultations

Multivariable models highlighted several consistent trends across the outcomes (Table 4). For nearly all outcomes, performance declined as health worker age increased, with about a five-percent decrease in the odds of recommended or adequate treatment per additional year of age (Fig. 2). A caseload of 10 or more children on the day of the child’s consultation was associated with a 33 % increased odds of recommended treatment. The number of supervision visits the health worker received in the previous six months was significantly associated with improved care for fever and anemia—although paradoxically associated with a lower odds of recommended treatment for pneumonia. For adequate fever treatment, the interaction between health worker age and the number of supervision visits received was significant, with more supervision visits attenuating the decline in health worker performance seen with increasing age. The figure in Additional file 2: Web Appendix 2 illustrates this interaction. Having at least one other IMCI-trained health worker working at the facility was significantly associated with better care for pneumonia, but not for other diseases. Health worker sex and facility type were not significantly related to any of the outcomes assessed.

**Table 4:** Predictors of ten health worker performance outcomes from multivariate analyses

	(1)	(2)	(3)	(4)	(5)	(6)	(7)	(8)	(9)
Variable	Recommended treatment	Adequate treatment	Adherence to guide-lines (%)	Recommended fever treatment	Adequate fever treatment	Recommended pneumonia treatment	Adequate pneumonia treatment	Recommended anemia treatment	Adequate anemia treatment
Mean value	63.6%	77.8%	96.5%	80.1%	87.5%	47.1%	75.6%	76.0%	82.7%
*Health facility level*									
Large (vs. small) facility									
*Health worker level*									
Health worker age, years	0.96**	0.93**	−0.11*	0.94**	0.89**	0.92**	0.95**		0.95
Female health worker							1.46		
10+ consultations on day of visit	1.33*			1.56**		1.18	1.59	1.58*	
Previous malaria training^†^			1.18			2.96*			
No. of supervision visits in previous 6 months	1.07		0.17	1.18**	1.13	0.87*		1.15*	1.15*
1+ other IMCI-trained health worker at facility	1.38					1.77*	1.90*		
*Patient level*									
Age <1 year	1.82**		0.66**	1.37*		1.68**	1.53**	2.63**	
Female sex		1.16			1.27*				
Patient temperature, degrees Celsius	0.96		−0.30**		0.88				
No. of total IMCI tasks required (case complexity)	0.95**	0.96	−0.51**					0.88**	0.99
At least one danger sign	0.33**	0.19**	−3.38**	0.08**	0.07**		0.45	30.52**	9.86**
Gold-standard diagnosis of fever		1.41	2.89**	^¥^	^¥^			4.24**	3.11**
Gold-standard diagnosis of anemia	0.27**	0.49**	−0.97**					^¥^	^¥^
Gold-standard diagnosis of pneumonia	0.83	0.61*	1.17**			^¥^	^¥^	2.03**	
Gold-standard diagnosis of diarrhea		0.39*	3.41**					3.28**	
Patient complaint of cough or difficult breathing	0.94	0.97	0.85*						
Patient complaint of diarrhea		2.04**							1.10
Patient complaint of fever or malaria			−0.30				0.78		1.76*
Interaction: HW age x no. of previous supervision visits					1.02*				

Note: each column contains the measure of association between the predictors included in a model and the outcome. All measures of association are odds ratios, except for outcome 3, which is the absolute percentage-point change in the outcome per unit change in the predictor). Blank cells indicate that predictors were not included in the model because they were neither significant in bivariate analyses nor a confounder/additional predictor (see Methodology section for further details).

*Significant at p<0.05 level

**Significant at p<0.01 level

^¥^Predictor excluded from the model due to collinearity

^†^Prior to IMCI training, which all health workers received. Previous diarrhea training modeled for diarrhea treatment outcome

At the patient level, infants received significantly better care across nearly all outcomes, with 1.82 times the odds of recommended treatment than older children. Case complexity was negatively associated with recommended care (overall and for anemia), as well as adherence to guidelines. Care was substantially and consistently worse for children with a danger sign, with the exception of anemia treatment, which was significantly better. One explanation of this finding could be that for febrile, respiratory, and diarrheal illnesses, children with any danger sign need referral and pre-referral treatments; in contrast, for severe anemia, no pre-referral treatment is needed according to the guidelines, and simple referral is sufficient. Additional file 3: Web Appendix 3 shows the 10 outcomes stratified by disease severity. For all three primary outcomes, care was significantly worse if the child had a gold-standard diagnosis of anemia (row 15 of Table 4). Unlike health worker age and presence of a danger sign, however, this association was only seen for one secondary outcome (diarrhea).

Findings for two disease-specific outcomes deserve particular attention. First, children with anemia who had another PLTI illness classification (fever/malaria, pneumonia, or diarrhea) were significantly more likely to be correctly treated for anemia than those with only an anemia classification (column 8 of Table 4). Presence of another illness might have prompted health workers to be more thorough in their treatment and, for fever/malaria, treatments for the two diseases overlapped. Second, having a patient complaint of diarrhea was associated with better diarrhea treatment (column 10 of Table 4), although patient complaints were not associated with better treatment for febrile or respiratory illnesses.

## Discussion

To understand what might have influenced health worker performance after IMCI training, we assessed a broad set of factors across the most common child illnesses in Benin. Mean health worker performance, though significantly improved after IMCI training, especially for health workers who received additional supports [20], was still insufficient, with only 63.6 % of children with PLTI receiving recommended treatment. Health workers performed thorough assessment and classification tasks, but fell short on providing recommended treatments to children. Our analyses showed that a range of factors were associated with case management quality, which has been seen in other studies of health worker performance [24–26]. In general, factors largely outside managers’ direct control, such as patient-level factors and health worker age, were the most important predictors of case-management quality. Factors more directly within program managers’ control, such as frequency of supervisory visits or in-service training, were found to have lesser effects on quality of care.

Increasing health worker age was associated with consistently worse performance (for 7/10 outcomes), suggesting that older health workers should be prioritized for quality improvement strategies. To our knowledge, only one previous study, also from Benin, found worse performance among older health workers [27]. Health worker age and experience were highly correlated in our analysis, and previous studies have found improved performance with greater health worker experience [28]. While it is possible that health worker age might be a proxy for another unmeasured characteristic besides experience, several plausible explanations exist for this finding. Older health workers, who also had more years of experience, may have relied more on their clinical experience and intuition and been less apt to follow guidelines, in line with other studies that have found that higher-level cadres of health workers tend to perform less well at following new guidelines than lower-level cadres [29, 30]. Alternatively, older health workers may be more likely to follow guidelines learned during initial pre-service or early in-service training, when they were more open to new ideas. It is also possible that older health workers were older than IMCI trainers and perceived the trainers to be less knowledgeable.

Children ages one to four years old received worse care than infants for most outcomes, an association found in another study of IMCI-trained health workers in Morocco [24]. Two other studies, neither of which were of IMCI-trained health workers, found worse care for infants [25, 31]. This finding, which perhaps results from the perception that younger children are particularly vulnerable, supports the importance during initial IMCI training and routine supervision visits of emphasizing the vulnerability of all children under five years.

Increased case complexity, defined as the number of IMCI tasks required, was associated with worse performance in terms of recommended treatment, adherence to guidelines, and anemia treatment. A likely explanation is that with greater case complexity there are more opportunities for errors. A good example of this is the low scores for recommended anemia treatment, which generally involves prescribing three different medicines (antimalarial, iron, and folate), all in correct doses. For more complex treatments such as that for anemia, more time during initial IMCI training and post-training supervision should be devoted to reviewing the treatment and reminding health workers to consistently prescribe all three medications.

Presence of a danger sign was consistently associated with worse care except for anemia treatment, for which it was associated with better care. Correct care for children with a danger sign involved referral plus any required pre-referral treatment, which was often skipped. As there is no pre-referral treatment for anemia, referral was sufficient.

We found a complex relationship between patient complaints and quality of care. In some cases, complaints probably prompted health workers to give a needed medicine—for example, antimalarials for children with anemia and ORS for children with diarrhea. This positive relationship was found in several previous studies of fever treatment [32–34]. However, there is not always a simple correspondence between a complaint and correct treatment. In our study, fever complaint was not associated with better fever care. Furthermore, patient complaints can paradoxically be associated with worse care for other diseases. For example, a complaint of cough or difficult breathing, which may have prompted health workers to zero in on pneumonia diagnosis, was associated with worse diarrhea treatment.

Significant predictive factors of health worker performance that are potentially more modifiable (e.g., through quality improvement interventions) were less consistently related to better case management quality. The number of supervision visits the health worker had in the past six months was positively related to several outcomes, including anemia treatment, but was associated with worse pneumonia treatment. One possible explanation is that the IMCI training and subsequent supervision was focused primarily on fever/malaria treatment, which was a larger perceived problem at the time in Benin. For adequate treatment of fever, additional supervision visits also appeared to attenuate the worse performance with increasing health worker age (see Additional file 2: Web Appendix 2). A review of the effects of supervision in low- and middle-income countries has found limited high-quality evidence and uncertain results of its impact [35].

Finally, higher patient volumes the day of the child’s consultation was associated with significantly better care for some outcomes. Higher volumes of IMCI patients perhaps afforded health workers more opportunities to practice their new skills, although we cannot rule out another underlying factor, given the small number of consultations (6.5 %) that took place on days where the health worker saw at least 10 IMCI patients. It should also be noted that the volumes in these facilities in Benin are on average quite low, and thus higher volumes in this setting would not overwhelm health workers or result in extremely limited consultation time as they might in other settings [27, 36].

### Strengths and limitations

This study has several important limitations. Although the dataset included a very large number of consultations, our sample size of health workers (n = 32) was relatively small, limiting our ability to assess in more detail health worker factors influencing performance. Second, our analysis relied on data abstracted from patient registers, which assumes that these registers accurately capture the patient consultation. However, completeness of register documentation was quite high, and 98.4 % of required IMCI assessment tasks were documented in the registers by health workers. Although we cannot know with certainty the accuracy of information in the registers, survey data from health worker observations and patient re-examinations from the same time frame in Benin showed similar trends as the register review data [20]. Therefore, in settings where health worker documentation has been assessed to be complete, and potentially where a sample of register data can be validated against health worker observations, register review data abstraction can permit analysis of a large number of consultations at lower cost than health worker observations and can also avoid the “Hawthorne” bias of better performance when health workers know they are being observed [26, 37–39]. However, particular attention should still be given to assessment of danger signs, which studies have indicated health workers might miss during assessments [40, 41].

Third, our analyses were more exploratory than confirmatory in nature, with multiple statistical tests performed across the 10 outcomes, thus increasing our chances of Type I error. Although we did not make any statistical adjustments (e.g., Bonferroni correction) for the multiple tests [42], we are relatively confident that relationships that held across several outcomes are not likely to be spurious. Fourth, it is possible that health worker practices were influenced by other factors that were not measured in this study, such as health worker motivation and beliefs in the importance of following guidelines [22]. Fifth, we were unable to assess the quality of counseling for patients, given that this was not documented in the register. Finally, our data are drawn from facilities in a small geographical area of Benin during a period immediately following initial IMCI introduction in the early 2000s, thus representing a relatively narrow context and highlighting the importance of confirming these associations through additional studies. Although the IMCI algorithm has been updated since this study, most notably to include diagnostic testing for malaria, health worker performance against IMCI guidelines is still low [23, 43] and understanding the factors influencing their behavior from this and other studies can help in crafting responses to poor performance—for IMCI and other guidelines [44].

## Conclusions

Despite its limitations, this study offers several important findings that have implications for case management in Benin and other settings. First, a range of factors at various levels can influence case-management quality, and many of these influences can be outside a manager’s direct control. For example, health worker age had one of the most consistently strong negative influences on case management quality. Although health worker age might be a proxy for another factor (e.g., experience or resistance to change) more directly related to poor performance, it is a measurable indicator and points to the importance of evaluating individual health worker performance on a regular basis and having targeted follow-up with poorly performing individuals.

Significantly poorer treatment for children with danger signs or anemia and some evidence of poorer treatment for more complex cases indicates potential gaps of IMCI training and supervision in addressing these types of patients. As real-life examples of children with danger signs or more complex patient presentations might be uncommon during initial IMCI practical training, IMCI program managers should take advantage of training modalities such as videos to expose health workers to more complex cases. In addition, supervisors should review documented cases of children with danger signs or anemia during supervision visits and refresher training and remind health workers about co-morbidities and the importance of assessing and addressing all signs and symptoms. This would also make consultations less driven by patient complaints, for which we saw some evidence, and more by comprehensive health worker assessment and classifications.

It is encouraging to see that additional supervision visits were associated with better performance across a number of outcomes, as previous evidence on supervision has been mixed [35]. A separate assessment found that the quality of supervision was generally good [45], and the supervision process was specifically tailored to improve performance for IMCI-recommended tasks. However, the low levels of performance of certain health workers (see Fig. 1), which did not change significantly over time [21], suggest that supervisors should focus more attention on underperforming health workers. In addition to more structured supervision, and regular on-the-job and refresher trainings, program managers should also consider creative strategies, such as group problem solving (including improvement collaboratives [46]) or text message reminders to health workers’ mobile phones [47, 48] or other forms of technology such as electronic job aids [49], to improve particularly challenging performance issues.

## Additional files

Additional file 1:
**Web Appendix 1: Photographs of modified IMCI patient register.**
Additional file 2:
**Web Appendix 2: Graph of interaction between health worker age and number of supervision visits and effect on adequate fever treatment.**
Additional file 3:
**Web Appendix 3: Outcome results stratified by patient severity (for any PLTI classification).**
