# Supplementary material for: Predictors of health worker performance after Integrated Management of Childhood Illness training in Benin: a cohort study
Source: BMC Health Serv Res. 2015 Jul 21;15:276. doi: 10.1186/s12913-015-0910-4 (PMC4509845; doi:10.1186/s12913-015-0910-4)
Supplement: Additional file 1: — Web Appendix 1: Photographs of modified IMCI patient register. [file 12913_2015_910_MOESM1_ESM.docx]

**Web Appendix 1**

Left page

Right page
