# Supplementary material for: Predictors of health worker performance after Integrated Management of Childhood Illness training in Benin: a cohort study
Source: BMC Health Serv Res. 2015 Jul 21;15:276. doi: 10.1186/s12913-015-0910-4 (PMC4509845; doi:10.1186/s12913-015-0910-4)
Supplement: Additional file 2: — Web Appendix 2: Graph of interaction between health worker age and number of supervision visits and effect on adequate fever treatment. [file 12913_2015_910_MOESM2_ESM.docx]

**Web Appendix 2: Graph of interaction between health worker age and number of supervision visits and effect on adequate fever treatment**
