# Supplementary material for: Predictors of health worker performance after Integrated Management of Childhood Illness training in Benin: a cohort study
Source: BMC Health Serv Res. 2015 Jul 21;15:276. doi: 10.1186/s12913-015-0910-4 (PMC4509845; doi:10.1186/s12913-015-0910-4)
Supplement: Additional file 3: — Web Appendix 3: Outcome results stratified by patient severity (for any PLTI classification). [file 12913_2015_910_MOESM3_ESM.docx]

**Web Appendix 3: Outcome results stratified by patient severity (for any PLTI classification)**

| **Outcome** | **Not Severe** | **Severe** |
| --- | --- | --- |
| Recommended treatment | 64.8 | 57.2 |
| Adequate treatment | 79.1 | 66.5 |
| Adherence to guidelines (%) | 97.6 | 95.3 |
| Recommended fever treatment | 81.8 | 30.4 |
| Adequate fever treatment | 89.1 | 41.9 |
| Recommended pneumonia treatment | 76.4 | 69.6 |
| Adequate pneumonia treatment | 81.6 | 80.6 |
| Recommended anemia treatment | 45.1 | 94.2 |
| Adequate anemia treatment | 75.2 | 94.2 |
| Recommended diarrhea treatment | 84.7 | 31.4 |
